# The Influence of Prior Knowledge on Memory: A Developmental Cognitive Neuroscience Perspective

**DOI:** 10.3389/fnbeh.2013.00139

**Published:** 2013-10-08

**Authors:** Garvin Brod, Markus Werkle-Bergner, Yee Lee Shing

**Affiliations:** ^1^Center for Lifespan Psychology, Max Planck Institute for Human Development, Berlin, Germany

**Keywords:** lifespan development, child development, hippocampus, medial prefrontal cortex, semantic memory, prior knowledge, lateral prefrontal cortex, episodic memory

## Abstract

Across ontogenetic development, individuals gather manifold experiences during which they detect regularities in their environment and thereby accumulate knowledge. This knowledge is used to guide behavior, make predictions, and acquire further new knowledge. In this review, we discuss the influence of prior knowledge on memory from both the psychology and the emerging cognitive neuroscience literature and provide a developmental perspective on this topic. Recent neuroscience findings point to a prominent role of the medial prefrontal cortex (mPFC) and of the hippocampus (HC) in the emergence of prior knowledge and in its application during the processes of successful memory encoding, consolidation, and retrieval. We take the lateral PFC into consideration as well and discuss changes in both medial and lateral PFC and HC across development and postulate how these may be related to the development of the use of prior knowledge for remembering. For future direction, we argue that, to measure age differential effects of prior knowledge on memory, it is necessary to distinguish the availability of prior knowledge from its accessibility and use.

## Introduction

As humans, we do not store verbatim copies of experiences in our memory. Rather, we integrate new incoming information from the surroundings in relation to our pre-existing knowledge about the world. This knowledge is accumulated across ontogenetic development through experiences during which the individual detects regularities in the environment. Growth in knowledge is one of the most prominent aspects in ontogeny and exerts its influence on memory functioning across the whole lifespan (Craik and Bialystok, 2006). The importance of prior knowledge for memory has been introduced in the classical work of Piaget (1929) and Bartlett (1932). Bartlett (1932) showed that humans, while recalling a specific event, often construct these memories based on their knowledge about the world, thus illustrating the susceptibility of human memories to errors due to their reconstructive nature. In his work with children, Piaget (1929) showed that, in addition to the assimilation of new information into existing knowledge frames (or schemata), knowledge has to be updated frequently in order to adapt to changing demands of the environment, a process he called accommodation. Despite the long-standing recognition of the important role of prior knowledge, most psychological and cognitive neuroscience experiments are designed with the implicit assumption that learning and memory take place in a tabula rasa state of the brain. So far, surprisingly little is known about how the interaction between pre-existing knowledge and new incoming information takes place within the brain.

In the following sections, we discuss both behavioral and neuroscience findings concerning the influence of prior knowledge on memory. We focus on studies that examined long-term storage of memory, as prior knowledge directly influences cognitive processes that are important for learning and retaining new information in the memory system. The representations built up from these processes form the basis of semantic memory, which is factual knowledge about the world, and episodic memory, which is memory bound in time and place (Tulving, 1972). In the remainder of the review, we outline a developmental cognitive neuroscience perspective that combines our understanding about changes in brain structure and function across development with behavioral findings of age differences in the use of prior knowledge for remembering. This developmental cognitive neuroscience perspective shall guide future investigations of age-related changes in the use of prior knowledge for remembering in brain and behavior simultaneously. Throughout the review, we use the term prior knowledge in a broad sense as stored knowledge and beliefs about the world that have been acquired by an individual. This knowledge can be declarative (i.e., semantic, episodic) or non-declarative (e.g., implicit or procedural). We hereby acknowledge that differences among related terms such as conceptual knowledge, rule knowledge, associative knowledge, and schema are not being considered.

## Behavioral Evidence Illustrating the Influence of Prior Knowledge on Memory

In a classical study, Bransford and Johnson (1972) demonstrated the impact of prior knowledge on comprehension and memory. In a series of experiments, participants were asked to listen to prose passages and were afterward tested on their memory for them. Participants who received relevant knowledge or cues before listening to the passages and therefore had prior knowledge available showed improved comprehension and better recall compared to participants who either did not receive cues or received the contextual knowledge only after hearing the passages. In a second experiment, Bransford and Johnson (1972) used descriptions of activities that were known to the participants. They manipulated whether the participants could access this knowledge by either providing or not providing a cue that allowed the activation of an appropriate context. As expected, comprehension and recall were better for the group that received the cue beforehand as compared to the no-cue group. In sum, the results indicate that if prior knowledge is available and accessible, it facilitates comprehension and memory of new incoming information. Moreover, these findings corroborate the view that our experiences are not remembered as exact copies, but are actively integrated into one’s existing knowledge structures.

In a similar vein, Craik and Lockhart (1972) argued that memory performance is not a simple function of the amount of encoded features (that is, the more, the better), but also of the qualitative nature of these features, i.e., how well they can be integrated into pre-existing knowledge. For example, Craik and Tulving (1975) demonstrated that words that were embedded in a congruent sentence context were better remembered than those that were embedded in an incongruent context (see also Schulman, 1974). In addition to showing a general benefit for congruously encoded items, Craik and Tulving (1975) also showed that a semantically rich context benefits memory for congruent words but does not affect memory for incongruent words. These results were taken to implicate a more elaborative encoding for congruent as opposed to incongruent information. Also, new information is more effectively integrated into existing semantic networks when presented stimulus and context form one unit. Since Craik and Tulving (1975), this so-called “congruency effect” has been replicated numerous times with different stimulus materials and tasks (e.g., Staresina et al., 2009; for a review, see Alba and Hasher, 1983).

Following the initial idea that prior knowledge benefits encoding via integration into existing semantic structures, Moscovitch and Craik (1976) provided a retrieval-related view on the importance of semantic context. In an incidental encoding task, participants read words on a screen and were asked to indicate whether those words either rhyme with another word, fit into a given category, or fit into a given sentence. The latter task was assumed to provide the deepest level of encoding. In addition, they manipulated retrieval conditions by irregularly presenting the initial encoding questions once more that did (yes-answers) or did not (no-answers) form a congruent unit with the target word. They showed that both cueing and congruency at retrieval enhanced recall and that this worked best in combination with deep levels of encoding. The results led the authors to postulate that recall performance depends on three factors: the quality of the trace (defined by the level of processing), the presence of retrieval cues, and the degree of congruity of the items with their context. Put differently, deep encoding comes along with a high potential of being remembered. The extent to which this potential is realized, however, depends on whether the retrieval context provides enough information to recreate the encoding context and on whether this context and the target stimulus form one unit. When all (or some) conditions are met, the retrieval of accurate targets will be facilitated.

Although it is well accepted that semantic congruency promotes memory performance, events that are incongruent to the prevailing context have also been shown to-be-remembered well. For example, the classical von Restorff-effect (Köhler and von Restorff, 1937) denotes that an item that is distinct from its surrounding items is more likely to-be-remembered than one that is not (also called “isolation effect”). Whether a given memory event profits from congruency or incongruency probably depends on several contextual features, such as the occurrence ratio between congruent and incongruent information (Alba and Hasher, 1983; Rojahn and Pettigrew, 1992). By taking a 50/50 ratio of congruent and incongruent stimuli, differences in saliency are reduced, which would be more prominent for, say, an 80/20 ratio in which the less frequent material type is expected to show isolation-like memory benefits (Hunt and Lamb, 2001).

However, explaining effects of distinctiveness with saliency or isolation alone is not sufficient. One intuitive mechanism through which distinctiveness operates is the effect of increased attention to the salient item, which is sparked by, for example, an emotional response to salience or surprise (Hunt and Lamb, 2001). Contrary to this explanation, it was shown that subjectively perceived salience is not necessary for the isolation effect (Dunlosky et al., 2000). Alternatively, distinctiveness can be understood as “the processing of difference in the context of similarity” (Hunt and Lamb, 2001). This implies that distinctiveness is not a property of the physical objects, but a certain kind of cognitive processing that creates more elaborate traces for isolated items. Accordingly, the end product of distinctive processing is an elaborate memory trace that is highly unique and easy to access during retrieval. Although this notion focuses on memory benefits due to the incongruity of an event, it is consistent with the general notion of the levels-of-processing account stating that remembering depends on the degree of elaboration of a trace during encoding in relation to existing knowledge structures (Craik and Lockhart, 1972).

### Conclusion regarding behavioral data

Prior knowledge facilitates processing of new incoming information, supposedly because it provides a structure into which the new information can be integrated, which may lead to an elaborated memory trace. This advantage of prior knowledge may hold, no matter if the new information meets expectations (is congruent with existing knowledge) or not (is incongruent). However, having prior knowledge available does not suffice, it needs to be accessed and used to benefit encoding (Bransford and Johnson, 1972; Alba and Hasher, 1983). Moreover, an elaborated memory trace only provides potential for later remembering. The extent to which this potential is realized might then depend on whether the retrieval context matches the encoding context to a certain degree and on whether it is distinct enough to activate the specific target trace. This suggests that it is necessary to look at retrieval as well in order to understand the memory benefit for elaborated information.

During retrieval, semantic knowledge might help to reinstate the encoding context, which was shown to be facilitated if the item is expected to occur in the specific context based upon prior knowledge (Moscovitch and Craik, 1976). From a spreading activation network perspective, an existing semantic structure provides a search space that is likely to contain additional routes by which the new information can be inferred if direct retrieval fails (Anderson, 1981). This may also be true if the item posits a mismatch with the context, as the degree of distinctiveness or novelty can also be diagnostic at retrieval. From a neuroscience point of view, however, only little is known about how these computations are carried out in the brain. In the next section, we will review recent neuroscience findings concerning the processing of information related to prior knowledge, particularly during memory processing.

## A Neuroimaging View on the Connection between Prior Knowledge and Memory

This section will first offer an overview over initial evidence suggesting the involvement of the medial temporal lobe [(MTL), especially the hippocampus (HC)] and PFC structures in the coordination of forming and applying knowledge. Based on this, we will examine the roles of MTL and PFC in making use of prior knowledge for the service of memory encoding, consolidation, as well as retrieval. A large part of this section will deal with medial prefrontal cortex (mPFC) and HC, as recent evidence and theorizing suggest both areas to be key regions for understanding the interplay between knowledge and memory functions. In addition, we will discuss the involvement of lateral PFC subregions. The latter provide important control and elaborative functions with regard to accessing and evaluating internal mnemonic representations.

The hippocampal formation forms part of the MTL and is a set of cortical regions comprising the dentate gyrus (DG) and the individual CA-fields in the HC as well as the subicular complex. The entorhinal cortex, perirhinal cortex, as well as the parahippocampal cortex, which all surround the HC, also play a role in the functioning of memory as they are the primary sources of neocortical inputs to the HC (Squire, 1992; Andersen et al., 2007). Regarding subregions of the mPFC that are presumably relevant to prior knowledge, we refer to Brodmann areas (BA) 12 and 25, the ventral parts of BA 32, and the medial parts of BA 10 and BA 11. Taken together, those subregions are similar to the subgenual ventromedial PFC (vmPFC) as defined by Nieuwenhuis and Takashima (2011), but also include anterior and dorsal parts of BA 10. There is a broad literature about mPFC function and anatomy in rodents. However, as this is not the main focus of the present review, the interested reader might refer to Nieuwenhuis and Takashima (2011) for an overview and comparison with the human mPFC.

### The emergence of knowledge from the coordinated interaction between mPFC and HC

Both MTL and PFC play a crucial role in the emergence and application of abstract knowledge, as demonstrated by Kumaran et al. (2009). In this study, participants played the role of weather forecasters and had to learn which patterns of associative visual stimuli predicted sun or rain. This task could either be solved by learning the concrete surface pattern or by abstracting the commonalities across relevant patterns. The latter would supposedly lead to the emergence of conceptual knowledge that would allow transfer to new situations. The degree to which the participants acquired abstract knowledge was tested with a transfer task. Here the same higher-order task structure was used, but with different patterns of visual stimuli, thus making it necessary for the participants to reactivate the abstract conceptual representation acquired during the learning phase. The gradual acquisition of knowledge that allowed successful weather predictions was positively correlated with activation in the mPFC, HC, and posterior cingulate cortex. Moreover, better knowledge about the hierarchical structure was associated with an increased functional coupling between the HC and mPFC. The transfer task revealed correlations between transfer performance and neural activity in the left HC. A subsequent study by Kumaran et al. (2012) corroborated those initial observations. There, the authors explored the formation of knowledge of social and non-social hierarchies and its impact on the ability to perform transitive inference (e.g., if A > B and B > C, then A > C). Again, activity increases in the posterior HC and the mPFC paralleled the emergence of hierarchy knowledge, independent of it being a social or a non-social hierarchy (Kumaran et al., 2012).

The HC has traditionally been implicated in the creation and retrieval of enduring episodic memory traces (e.g., Simons and Spiers, 2003). Along this line, the role of specific HC-subfields (e.g., the DG) in the orthogonalization of representations of similar input patterns has been highlighted (i.e., pattern separation; McClelland et al., 1995). More recent studies also demonstrate HC involvement in inferential processing, such as flexibly combining memories to allow knowledge transfer (Zeithamova and Preston, 2010). Generalization across episodes might be supported via recurrent processing (i.e., pattern completion), either within specific HC-subfields (i.e., CA3/CA1; Marr, 1971; Treves and Rolls, 1992) or in larger HC-entorhinal cortex loops that act upon orthogonalized representations (for an in depth overview, see Kumaran and McClelland, 2012). Hence, a crucial facet of efficient HC processing resides in maintaining a fine balance between pattern separation and pattern completion operations (Yassa and Stark, 2011). These postulations still need to be validated by empirical evidence but might give a hint on how the HC is involved in constructing new abstract knowledge and how hippocampal computations may be susceptible to attentional modulation, possibly from the PFC (Duncan et al., 2009).

The mPFC, in turn, has traditionally been implicated in various functions including self-referential processing (e.g., Northoff and Bermpohl, 2004) and processing of reward-related information (e.g., Behrens et al., 2008). Given its involvement in reward processing, Kumaran et al. (2009) suggested that the mPFC may guide decision-making by integrating information received from the HC (discrete memories of encountering specific associative visual stimuli) with its associated value information (e.g., correctness of prediction, gain, and losses).

It is important to note that actual knowledge is probably not stored within the mPFC or the HC. In spite of the rich literature on representation of knowledge in the brain, we will limit ourselves to the statement that the storage of human knowledge corresponds to a network of parietal and temporal heteromodal association areas that receives input from multiple modalities (for review and meta-analysis, see Binder et al., 2009; Binder and Desai, 2011). HC and mPFC are suggested to form a network that builds up and integrates associative information with valuation, which then guides the acquisition of new conceptual knowledge (Kumaran et al., 2009). So far, the exact mechanisms through which the HC, mPFC, and parietal/temporal association areas interact remain unclear.

In the subsequent section, we will discuss how the HC and the mPFC are involved in memory processes, starting with encoding and retrieval and followed by the consolidation of memories.

### The effects of prior knowledge during memory encoding: The roles of mPFC and hippocampus

The initial findings by Bransford and Johnson (1972) that prior knowledge boosts comprehension and memory have been corroborated by neuroimaging work that tried to identify the neural correlates of this enhancement. An early PET-study (Maguire et al., 1999) adapted the paradigm of Bransford and Johnson (1972) by providing participants with relevant, irrelevant, or no visual cues before listening to stories in which the storyline was difficult to grasp. Maguire et al. (1999) revealed activations in the dorsal posterior cingulate area (PCC, BA 31) that was related to hearing the unusual passage when the helpful context picture was presented beforehand. Participants’ ratings of the comprehensibility of the stories were positively correlated with activation in BA 31 and in ventral medial orbitofrontal cortex (BA 11) while activation in the left middle frontal gyrus (BA 10) was correlated with actual memory performance. These early findings emphasize the importance of prior knowledge for comprehension and memory by showing increased neural activations in medio-frontal regions (BA 10, BA 11) when knowledge is available during learning of new information.

More recent fMRI work examined effects of prior knowledge directly during episodic memory encoding. In a study by van Kesteren et al. (2010a), the availability of prior knowledge was manipulated by exposing two groups of participants to the first 80 min of a movie, either in correct (consistent schema) or scrambled order (inconsistent schema). On the next day’s fMRI session, participants watched the movie’s final 15 min in original order, after which they stayed in the scanner for an additional 15 min resting period (administered 10 min after encoding). Participants with an inconsistent schema showed higher correlations between HC- and mPFC-activity and less mPFC intersubject-synchronization, a measure of across-subject BOLD signal coherence. The higher correlation between HC and mPFC in the inconsistent schema group was interpreted as compensatory connectivity in order to make up for their lack of consistent schema. Interestingly, this increased correlation persisted during the 15 min post-encoding resting period, suggesting that a lack of prior knowledge could have resulted in increased spontaneous replay of the newly encoded information.

In a subsequent study, van Kesteren et al. (2013) assessed the influence of subjectively perceived congruency on mPFC and HC activation. In the MRI scanner, participants rated the congruency of object-scene pairs (e.g., classroom – chalk). About 24 h later outside of the scanner, they were tested on item and associative memory of the pairs. van Kesteren et al. (2013) examined the so-called subsequent memory effect, in which brain activations from encoding trials that resulted in subsequent remembering are directly contrasted with trials that are subsequently forgotten. The mPFC displayed an increase in the subsequent memory effect with congruency, whereas the left parahippocampal cortex showed a decrease with congruency in the subsequent memory effect. These findings support the notion that the mPFC plays a key role in the integration of new congruent information, whereas MTL areas are involved during the encoding of incongruent information (van Kesteren et al., 2012).

Taken together, initial evidence points to an involvement of mPFC and MTL regions during the encoding of new information that can be related to prior knowledge. In the next section, we will expand on these findings and examine the role of mPFC and HC in memory consolidation and retrieval.

### The involvement of mPFC and HC in memory consolidation and retrieval

The importance of prior knowledge for memory consolidation was emphasized in a recent review by Wang and Morris (2010). The authors argue that the brain stores associative frameworks of knowledge, which are supposedly implemented as networks of interconnected neocortical representations. The dynamic build-up of such structures is made possible by activity-dependent synaptic plasticity, as well as dendritic and synaptic growth. Hence, setting up such associative knowledge structures takes time. If these structures exist, however, the assimilation of related new information is expected to be facilitated, which would lead to speeded consolidation.

This reasoning is backed by studies with rats (Tse et al., 2007, 2011; McKenzie et al., 2013). Tse et al. (2007) showed that the removal of the entire HC as early as 48 h after the rapid learning of two new flavor-place associations fully spared memory when the rats were given extensive pre-training on six other flavor-place associations, i.e., prior knowledge. Consistently, rats that lacked prior knowledge displayed severe memory distortions after HC removal. These results suggest that neocortical sites are capable of rapid associative learning, if relevant prior knowledge is available. Moreover, in a subsequent study, Tse et al. (2011) provided evidence that the shift of the indexing function from the HC to the mPFC is not just a shift of locus, but rather a development through concurrent HC-mPFC interactions [see initial evidence for rapid memory consolidation in humans in Takashima et al. (2009)]. In addition, McKenzie et al. (2013) showed that neurons in the rat HC that were specific to certain trained goal locations in a circular track were initially active during the learning of new goals as well. As learning progressed, however, hippocampal activity patterns for old vs. new goal locations gradually diverged from one another. These findings were taken to suggest that consolidation involves both assimiliation of new information into existing knowledge structures and accommodation of these structures to ensure accurate memories, and that the HC plays a role in these processes.

In studies with human participants, tracking consolidation processes in the brain via the use of neuroimaging techniques has become increasingly prominent. Takashima et al. (2006) assessed changes in neural activation elicited by the retrieval of visual stimuli in a 3-month period after initial learning. Over the course of the entire study, participants showed a decrease in HC activation and an increase in mPFC-activity for confidently recognized pictures (see also Yamashita et al., 2009). These findings suggest that the mPFC takes over linking functions from the HC for retrieving coherent remote memories (see also Yamashita et al., 2009). This reasoning is in accordance with an extended version of system consolidation theory, which states that, at first, the HC is necessary for storage and recovery of a memory trace. As consolidation proceeds, however, HC contributions diminish and cortical structures suffice to maintain the memory trace and to mediate its retrieval (Frankland and Bontempi, 2005; cf. Gais et al., 2007).

On the retrieval side, van Kesteren et al. (2010b) examined the neural underpinnings of the congruency effect during retrieval 24 h after learning in a visuo-tactile learning paradigm. Word-fabric combinations, which were either congruent or incongruent with common knowledge [e.g., the word “tie” was presented with a tie (congruent) or with a rubber (incongruent)] had to be associated with visual motifs. It was found that activity within the mPFC and the somatosensory cortex as well as the connectivity between the two areas was enhanced when motifs and associated words could be retrieved correctly. This was not the case when only the motifs were recognized without successful associative retrieval. Moreover, the increase in functional connectivity was positively correlated with the behavioral congruency benefit (i.e., more congruent hits than incongruent hits) associated with pre-existing knowledge across participants.

The finding of greater mPFC-activity for correctly remembered congruent motifs matches the supposed role of the mPFC in retrieval monitoring as providing a “feeling of rightness” for memory cues during retrieval. This monitoring function of the mPFC is assumed to bias later processing in the limbic system, including the HC (Moscovitch and Winocur, 2002). The existence of such a “feeling of rightness” is based on the compatibility of the memory cues with prior knowledge and is missing in confabulating patients with lesions in the mPFC (Gilboa et al., 2006). In line with this claim, in a recent review by Nieuwenhuis and Takashima (2011), it was argued that the mPFC integrates information from the limbic system and subsequently suppresses representations therein (especially in the HC) that might not be needed for retrieving information that fits prior knowledge. Based on this role of the mPFC, finding greater mPFC involvement during the retrieval of congruent compared to incongruent information seems conceivable, as only the former type of information elicits a “feeling of rightness.” However, thus far the hypothesis of less hippocampal activation during the retrieval of congruent stimuli (due to suppression from mPFC) has not received empirical support. Hippocampal activity for congruent and incongruent stimuli did not differ during retrieval (van Kesteren et al., 2010b).

Thus far it appears that the neural structures mainly associated with system consolidation, i.e., MTL and mPFC, are also involved in the formation and application of abstract knowledge. This may not be a coincidence, as the effect of consolidation and the effect of knowledge abstraction, i.e., forming a conceptual “gist,” resemble each other (cf. Ellenbogen et al., 2007). However, our remembering of remote episodes can certainly entail contextual details. In this case, evidence suggests that the HC remains involved (Nadel and Moscovitch, 1997).

### Lateral PFC contributions to the effects of prior knowledge on memory

Despite the key role of the mPFC in recent literature on memory and knowledge, earlier studies have also demonstrated the involvement of lateral parts of the PFC (lPFC) in memory processes related to knowledge use, such as semantic elaboration (e.g., Kapur et al., 1994; Wagner et al., 1998) and relational processing of features contained in the to-be-remembered information (e.g., Fletcher et al., 2000; Addis and McAndrews, 2006; Murray and Ranganath, 2007). An early PET-study based on levels-of-processing ideas showed that elaborative encoding, as compared to shallow perceptual processing, goes along with increased activity in the left inferior frontal gyrus (Kapur et al., 1994). In line with that, Wagner et al. (1998) found that activations in the left lateral inferior frontal gyrus (together with left parahippocampal and fusiform gyri) was higher for subsequently remembered than for forgotten words. This finding indicates that elaborative processes involving the left inferior frontal gyrus directly benefit memory. Furthermore, a study that manipulated encoding through instructions to either remember or forget the preceding stimulus revealed that the condition in which participants had the intention to encode was linked to an increased activity in the left inferior frontal gyrus (Reber et al., 2002). Finally, a recent study by Staresina et al. (2009) showed that congruent events (a match of word/color combination during encoding, e.g., the word “balloon” in front of a yellow background) yielded greater activation in the left lateral inferior frontal gyrus than incongruent events (e.g., the word “elephant” in front of a red background). Activation in the lateral inferior frontal gyrus was stronger for later remembered than for forgotten trials, indicating that its involvement in the task is predictive of memory performance. Taken together, the inferior frontal gyrus may be critically involved in semantic processing of incoming information, which leads to better episodic memory.

Besides the left inferior frontal gyrus, the dorsolateral PFC is contributing to the effects of knowledge on memory as well, supposedly due to its role in building relationships between items (Murray and Ranganath, 2007). In Murray and Ranganath’s (2007) study, participants saw sequentially presented unrelated word pairs and did either have to make a judgment concerning the relationship between the two words or concerning specific semantic attributes of the second word. Encoding the word pairs in relation to each other lead to a better recognition of the word pairs in an associative memory test. Activity in the dorsolateral PFC was greater in the relational judgment condition compared to the item-specific condition. Furthermore, activity in the dorsolateral PFC predicted performance in the associative memory test. These findings lead the authors to suggest that the dorsolateral PFC is involved in the active processing of semantic relationships (Murray and Ranganath, 2007).

In addition to semantic elaboration and relational processing, the lPFC is also heavily involved in memory control processes including monitoring. Memory control processes are crucial for evaluating representations retrieved from the HC in the context of current task goals, thereby allowing memory to be adaptive, in particular whenever the retrieved representations resemble each other or are both familiar (e.g., Ranganath et al., 2000; Mitchell and Johnson, 2009).

Taken together, findings on the involvement of the lPFC in memory processes related to knowledge use suggest that the lPFC is linked to intentional memorizing processes including semantic elaboration and relational processing. Given the literature discussed above that suggests lPFC involvement in a number of memory processes related to knowledge use, we argue for the need to integrate the lPFC into the picture when dealing with the effects of prior knowledge on episodic memory. We will revisit this issue in the following sections.

### Conclusions regarding neuroimaging data

Recent neuroimaging findings suggest that both mPFC and MTL regions (particularly the HC) contribute to the formation and utilization of knowledge and that they do so in an interactive fashion. During the formation and application of complex conceptual knowledge, it is proposed that the mPFC and the HC interact in a way that the HC detects regularities across episodes, which are integrated with value information (such as gains and losses, as well as emotional valence) by the mPFC (Kumaran et al., 2009). Along similar lines, Nieuwenhuis and Takashima (2011) proposed that the mPFC integrates and weights information associated with discrete episodes from the limbic system, namely the HC, the amygdala, and the ventral striatum.

The neural correlates of memory processing in relation to prior knowledge also involve a network comprised of HC and mPFC. According to the model of van Kesteren et al. (2012), the HC is involved in the detection and encoding of novel information and information that is incongruent to the encoding context. The mPFC deals with relating and integrating the incoming information to the existing knowledge base. It acts like a resonance detector, in the sense that information congruent to prior knowledge resonates with existing information. HC and mPFC processes, however, do not work independently of one another. It is assumed that the HC encodes new experiences when novelty is high. When the mPFC detects resonance, it will inhibit (or compete with) the HC, as the new information can also be encoded via relation to prior knowledge. Therefore, examining the interactions between MTL and mPFC appears critical for understanding the effects of prior knowledge on memory. While the model has received initial support especially concerning the role of the mPFC in prior knowledge effects on memory, it has to be noted that the role of MTL regions in memory processing of congruent/incongruent information and its relation to mPFC is less clear and requires further consideration and validation.

In addition to the mPFC, we also discussed lPFC involvement in a number of memory processes related to knowledge use and argued for the need to integrate the lPFC into the picture when dealing with the effects of prior knowledge on memory. However, it is currently unclear how the medial and lateral parts of the PFC differ in their contribution to knowledge-related memory processes. The only explicit model available at the moment is one that focuses on neural correlates of the predictive function of memory that is based upon prior knowledge (Kroes and Fernández, 2012). In this framework, the mPFC is assumed to contribute to the formation of complex episodic memories and abstract knowledge, while the lateral PFC contributes to simple rule learning. Concisely, the lPFC is suggested to interact with the lateral/inferior temporal cortex and to apply strict stimulus-response rules, whereas the mPFC acts in concert with the HC to allow predictions based on abstract knowledge transferable to new situations. These postulations will need to be tested empirically.

Observing the involvements of mPFC and HC in both the formation of knowledge and in the use of knowledge for memory, it is now tempting to ask (a) to what extent the localizations of mPFC and HC observed in these processes are overlapping within the same person, and (b) how the mPFC-HC activations/interactions observed in the building up of knowledge are related to the subsequent use of this knowledge in service of memory. These questions call for a study to track the building up of knowledge and the use of knowledge for memory within the same participants.

From a developmental viewpoint, the differentiation between medial and lateral parts of the PFC in relation to knowledge-related memory processing is very important. As we shall discuss in the sections below, the development of the effects of prior knowledge on memory have mostly been linked to functional and structural changes in the lPFC. Given that some regions within the mPFC also display a more protracted maturation trajectory (Shaw et al., 2008), there are potentially important changes within the functioning of the mPFC which support the above mentioned development by using prior knowledge in service of memory functioning.

## The Developmental Cognitive Neuroscience Perspective

Knowledge accumulates in the course of life through experiences during which the individual perceives and internalizes patterns in his or her environment. As the growth of knowledge is especially striking in early life, taking a developmental cognitive neuroscience approach with a focus on child development offers the unique possibility of exploring the effects of an expanding knowledge base on memory (Baltes et al., 2006; Craik and Bialystok, 2006).

First, we will provide an overview of general theorizing about developmental changes in cognition across the lifespan. Second, we will focus on how memory development is shaped by lifespan changes on a neural and behavioral level. For the discussion of developmental changes in the neural correlates of memory with age, we will focus on both structural and functional development of PFC and MTL and link changes in these areas to age differences in the use of prior knowledge for memory. We will expand on this topic by highlighting parallel changes in the knowledge base, which speaks to the issue of differences in the use of prior knowledge with age. As we will show, these parallels are particularly salient during child development, as childhood is the most dynamic period of knowledge development (e.g., Li et al., 2004).

Hence, in the present review, the lifespan perspective provides the frame for conceptualizing the specifics concerning the influence of an emerging knowledge base on memory during childhood. We will argue that, to measure age differences in the effects of prior knowledge on episodic memory, it is necessary to distinguish the availability of prior knowledge from its accessibility.

### General conceptions of lifespan changes in cognition

Knowledge is known to increase strikingly during childhood, to continue to accumulate throughout adulthood, to remain rather stable in old age, and to only decrease in very old age (Baltes, 1987; Li et al., 2004; Craik and Bialystok, 2006). As emphasized by Craik and Bialystok (2006), however, knowledge does not act independently. Even if there is no decrease in available knowledge with age, aging goes along with difficulties to access this knowledge. Older adults often express problems naming known objects even though, in principle, the names are available to them. This impairment in older adults could be linked to a temporary inability to access their knowledge, which can be overcome by giving appropriate cues or by offering more time (Cohen and Burke, 1993; Hasher et al., 2001). This nicely illustrates that knowledge can be available, but not accessible. In the framework offered by Craik and Bialystok (2006), the decrease in the ability to access knowledge is subsumed under the term cognitive control, which is known to increase steeply from infancy to young adulthood, and to decline thereafter (Bunge et al., 2002; Diamond, 2002; Zelazo et al., 2004).

The framework by Craik and Bialystok (2006) resembles the two-component model of lifespan cognition by Baltes et al. (2006). With regard to intellectual functioning across the lifespan, Baltes and colleagues distinguish between the mechanics and the pragmatics of cognition. The former are closely associated with the biological brain status, known to increase until early adulthood, and to decrease constantly thereafter. The latter are associated with the knowledge base, which is shaped by the socio-cultural environment. The cognitive pragmatics are shown to increase well into adulthood and to remain relatively stable until old age (Li et al., 2004). These comprehensive frameworks of cognitive change across the lifespan support the claim that the availability of knowledge on the one hand, and control processes allowing access to this knowledge on the other hand, follow strikingly different lifespan trajectories. Hence, when comparing age groups across the lifespan regarding a specific domain such as episodic memory, the common and unique contributions of cognitive mechanics and pragmatics need to be taken into account.

### Conceptions of lifespan changes in episodic memory and the need to integrate the prior knowledge perspective

Grounded in the comprehensive treatments of lifespan cognitive development, Shing, Werkle-Bergner, Lindenberger and colleagues [Shing et al. (2008, 2010); Werkle-Bergner et al. (2006)] proposed a framework that distinguishes two components to account for changes in episodic memory across the lifespan: an associative component, which refers to mechanisms of binding different features of a memory episode into a coherent representation, and a strategic component referring to control processes which aid both encoding and retrieval. The two components are assumed to interact and to differ in terms of their lifespan trajectory. The associative component, which is linked to the development of the MTL, is proposed to reach its high functionality already in middle childhood. The strategic component, which is linked to the development of the PFC, is assumed to show a protracted development and to increase in its functionality until young adulthood. Both components are hypothesized to undergo senescent decline in late adulthood and old age. Therefore, children’s difficulties in episodic memory performance are linked to immature strategic operations, whereas deficits among older adults are linked to impairments in both associative and strategic operations (Werkle-Bergner et al., 2006; Shing et al., 2008, 2010).

So far, there is no explicit handling of the general knowledge base’s lifespan changes in relation to the associative and strategic components of memory development. Nevertheless, understanding lifespan changes of the general knowledge base may contribute to an improved understanding of memory development. Initial evidence for this view comes from research on expertise and memory performance. Schneider et al. (1993) compared children and adults with both high and low chess expertise. The children and adults with high expertise remembered chess positions comparably well and much better than children and adults with low expertise. In a control digit span task, adults outperformed children, independent of chess expertise. This lead the authors to conclude that a rich knowledge base of a specific domain strongly affects memory for newly learned information within that domain and can even lead to a reversal of typical age trends.

Relating these findings to the two-component framework of lifespan changes in episodic memory (Shing et al., 2008, 2010), one could argue that prior knowledge exerts its influence on the strategic component only, as controlling for strategic operations attenuates performance differences between children and young adults (Brehmer et al., 2007; Shing et al., 2008). Indeed, as discussed above, PFC-driven strategic encoding and retrieval operations such as elaborative encoding or explicit memory search play a role in the observed memory benefits (Craik and Tulving, 1975; Anderson, 1981), presumably via affecting the accessibility of prior knowledge. Recent findings from neuroimaging, however, revealed joint changes in mPFC and HC activation and in the connectivity between the two that underpin the emergence and application of prior knowledge (Kumaran et al., 2009, 2012; van Kesteren et al., 2010a,b). Furthermore, system-level consolidation was shown to be facilitated when relevant prior knowledge was available (Tse et al., 2007). These findings suggest that prior knowledge might do more than just influence PFC-driven strategic operations; they suggest that prior knowledge drives the interaction between PFC and MTL regions, possibly leading to more efficient learning and consolidation processes (van Kesteren et al., 2010a,b). These novel findings call for further theoretical specification and empirical validation of the two-component framework incorporating knowledge base as a possible factor driving the interaction between the strategic and the associative component.

### Development of neural correlates of memory during childhood

As discussed above, MTL and PFC regions are crucially related to (a) the formation and application of knowledge and (b) episodic memory functioning. As apparent from structural neuroimaging work on brain development, these regions exhibit differential developmental trajectories. While maturation takes longest in prefrontal and parietal areas, the MTL as a whole does not show large structural changes during early and middle childhood, even though this might be different for some subregions (Sowell et al., 2003; Gogtay et al., 2006; Lavenex and Lavenex, 2013). The mPFC seems to display a more complex maturation trajectory that differs between the subregions (Shaw et al., 2008). Concisely, the orbital and posterior parts (BA 25, BA 32, posterior parts of BA 12 and BA 11) of the mPFC follow an early maturation pattern, whereas its anterior and dorsal parts (BA 10 and anterior parts of BA 12 and BA 11) follow the trajectory of the lateral PFC, which is late maturing (Shaw et al., 2008).

These structural findings suggest that functions associated with the PFC (i.e., strategic/control processes) might develop more slowly than the ones associated with the MTL (i.e., associative processes). This idea is supported by a study in which the subsequent memory paradigm was used to reveal activations associated with successful remembering (Ofen et al., 2007). Ofen et al. (2007) showed that activation for later remembered scenes in contrast to forgotten scenes increases with age in the PFC, but not in the MTL (see converging behavioral findings from Brehmer et al., 2007; Shing et al., 2008).

There is, however, also evidence for continued functional development in MTL regions until early adolescence (Ghetti et al., 2010). In Ghetti et al.’s (2010) study, children (aged 8–11), adolescents (aged 14), and young adults were given an incidental encoding task in which they saw colored drawings and had to decide whether the depicted object could be found in a house or whether the object was animate. Later, in a surprise recognition task, participants were asked to state whether they had seen the drawing in the scanner and, if so, in what color. For this detail recollection task, adults and adolescents engaged regions of the HC and of the posterior parahippocampal gyrus, whereas children did not. This study differs from Ofen et al. (2007) as it entails a greater need for recollection processes due to the requirement of remembering contextual details of the encoding episode (the color of the drawing, which was randomly assigned). Therefore, age differences in MTL involvement in an episodic memory task might also be dependent on task factors such as the demand for associative binding. Whereas MTL regions can therefore be considered critical for the formation of new episodic memories, their role for the acquisition of knowledge is less clear. An early study by Vargha-Khadem et al. (1997) revealed that early hippocampal damage does not preclude the acquisition of new knowledge, as their patients, despite suffering from damage to the HC from early age on, showed average performance in tests of factual knowledge.

In addition to changes in MTL and PFC regions, age-related changes in brain areas specialized for specific domain knowledge may come along with age differences in memory as well (Ofen, 2012). Evidence can be gathered both from behavioral studies on the influence of growth in knowledge base on memory (reviewed in the next section), and from recent neuroimaging findings. These studies revealed prolonged maturation in brain areas processing specific domain knowledge and linked this maturation to increases in memory performance (Golarai et al., 2007; Chai et al., 2010). By comparing children, adolescents, and young adults, Golarai et al. (2007) showed that the right fusiform face area and the left parahippocampal place area, two functionally defined areas important for faces and places, showed a substantial age-related increase in size. Moreover, this increase was correlated with improved recognition memory for faces and places. In a similar vein, a subsequent study by Chai et al. (2010) showed that the age-related expansion of the parahippocampal place area is correlated with better memory for complex scenes in participants aged 8–24.

In sum, brain regions that underpin memory differ regarding the time course during which they develop. While MTL regions are relatively mature already during middle childhood (but see Ghetti et al., 2010), the lPFC and parts of the mPFC show a protracted development which continues until late adolescence/young adulthood. Taking into account brain areas that are specialized for specific domain knowledge adds to this pattern as those areas display a prolonged maturation that could be related to memory performance. While the distinction between influences of PFC- and MTL-development on memory performance has recently gained considerable attention (see e.g., Ofen et al., 2007; Ghetti et al., 2010; Shing et al., 2010), future research will have to take the development of domain-specific areas into account as well.

### Development of knowledge and its relation to the neural correlates of memory

As discussed thus far, more elaborated semantic networks contribute to memory improvements with age. In accordance with this, children’s episodic memory has been shown to be influenced by their semantic knowledge about the to-be-remembered stimuli. For example, in an early study by Schneider et al. (1989), children (third, fifth, and seventh graders) who possessed a broad knowledge of soccer showed better recall of a soccer story than children that did not possess such soccer-related knowledge.

Earlier behavioral studies that assessed congruency effects in children of different ages revealed an age-related increase in the tendency to remember congruent information as opposed to incongruent information (Geis and Hall, 1978; Ghatala et al., 1980, for a meta-analysis see Stangor and McMillan, 1992). For example, in Ghatala et al. (1980), children aged 8–14 answered questions about 36 words that were either congruent with the questions (yes-answers) or incongruent (no-answers) and had to recall the words afterwards. A linear increase in recall accuracy with age was found for the congruent condition, whereas no change in recall accuracy with age was found for the incongruent condition. This increase in congruency effect with age was interpreted based on the levels-of-processing framework (Craik and Lockhart, 1972): although all words can be understood by all participants, older children have more opportunities to elaborate on the to-be encoded word because semantic knowledge grows with age. Ghatala et al. (1980), however, acknowledge that their findings are also consistent with a retrieval-related interpretation. This interpretation would suggest that older children engage more in strategic retrieval and might use the encoding questions as cues during recall, which is easier if the word matches its question (i.e., is congruent).

In a study with children aged 8–11, Maril et al. (2011) used the semantic congruency effect to manipulate the accessibility of prior knowledge in an item-color pairing paradigm, in which subjects had to decide whether a word/color combination was plausible. In an fMRI-analysis which took into account congruency as well as age, Maril et al. (2011) showed that adults rely more on structures in the parietal cortex and in the left lPFC (which, as mentioned before, can be linked to semantic processing), whereas children recruit more posterior brain areas (i.e., the right occipital cortex) associated with perceptual processing. Based on these findings, Maril et al. (2011) suggest that children may initially depend more on posterior perceptual systems in service of memory functioning, and, with age, develop more elaborative (semantic) knowledge structures. This extensive semantic knowledge base is then used for a more elaborative encoding, which, as shown by a main effect of age, is generally beneficial for memory.

In sum, Maril et al.’s (2011) study on the neural correlates of an age-related increase in the congruency effect suggests a rise in the use of semantic knowledge structures for remembering (Maril et al., 2011). This might go along with a decreasing importance of mere perceptual encoding, as indicated by a posterior-to-anterior shift in brain activation, and with an increasing importance of PFC-driven strategic encoding. This reasoning is in accordance with the developmental trajectory of gist vs. verbatim knowledge as proposed by Brainerd et al. (2004), a notion that we will turn to in the next section. Thus far, however, most developmental studies have not disentangled age-related differences between the availability and the accessibility and use of prior knowledge. We will discuss these issues in the summary section.

### The flipside of knowledge development

Does the accumulation of knowledge in the course of life improve memory performance in all situations? Evidence against this view is provided by research on false memory. In the DRM-paradigm (Deese, 1959; Roediger and McDermott, 1995), words that semantically converge to a common theme are presented during encoding. Later at recognition, participants are tested on their memory for the words studied beforehand. In addition, semantically related words never studied at encoding (*critical lures*) are also presented and participants are asked to reject those lures. In adult participants, the probability of falsely endorsing the critical lures is as high as that of the presented items. False recognition of semantically related words increases during childhood (Metzger et al., 2008; Paz-Alonso et al., 2008). Using the DRM-paradigm, Paz-Alonso et al. (2008) showed a correlation between the age-related increase in false alarms to critical lures and activation changes in the left ventrolateral PFC, which has been shown to be important for semantic elaboration (Wagner et al., 1998).

The DRM-paradigm illustrates that the more elaborate semantic knowledge structures of older children may improve the extraction of gist-like traces as opposed to the less semantic processing of younger children. This in turn leads to a higher likelihood of endorsing critical lures in older children (for a similar argument, see Smith and Hunt, 1998). Similar findings could also be revealed in an induction task (Sloutsky and Fisher, 2004) in which category (semantically)-based induction and similarity (perceptually)-based induction were disentangled. Adults typically perform induction in a category-based (semantic) manner, whereas children rely more on similarity-based (perceptual) induction. Category-based induction led to little discrimination between items presented during the induction task and lures that belonged to the same semantic category (e.g., another exemplar of the category cat). Similar to the findings using the DRM-paradigm, children displayed a higher memory accuracy compared to adults, which was due to a lower false alarm rate. Accordingly, training children to perform category-based induction lead to a memory performance comparable to the one of adults (Sloutsky and Fisher, 2004). These results show that relying on prior knowledge is not always beneficial for episodic memory performance. In specific situations where perceptual information is important, a reversal of typical age effects, i.e., children outperforming adults, can be found. This is due to the children’s stronger reliance on perceptual as compared to semantic processing, which is more prone to false memories because of overgeneralization.

## Summary and Open Questions

In this review, we discussed the influence of prior knowledge on memory considering both the psychology and the cognitive neuroscience literature. We reviewed classical psychology experiments that demonstrate the impact of prior knowledge on remembering new information (Bransford and Johnson, 1972; Craik and Tulving, 1975). We integrated the emerging cognitive neuroscience perspective on this topic, which points to PFC and MTL regions displaying activity changes as a function of prior knowledge. More specifically, recent studies suggest a prominent role of the vmPFC and the HC in underpinning the formation and application of prior knowledge (Kumaran et al., 2009, 2012; van Kesteren et al., 2010a,b). Both areas were shown to play a key role in consolidation as well. This is a conceivable idea given that the effect of consolidation and the effect of knowledge formation and application resemble each other, i.e., forming of a conceptual “gist” which might later be applied as a search frame during retrieval. Moreover, recent research indicates an activity increase in the vmPFC during post-consolidation periods (e.g., Takashima et al., 2006), which may occur earlier if new information can be assimilated into existing knowledge structures (Tse et al., 2007).

In addition, we outlined a developmental cognitive neuroscience perspective, considering changes in brain structure and function across child development and linking those changes to behavioral research on age differences in the influence of prior knowledge on memory. To conclude, we will now outline open questions and possible confounds regarding the assessment and interpretation of age-related changes in the use of prior knowledge for remembering.

First, to assess age differential effects of prior knowledge on episodic memory in an age comparative setting, we postulate that it is necessary to distinguish the availability of prior knowledge from its accessibility and use. As mentioned above, one of the most prominent changes in human ontogeny is growth in knowledge, that is, an increase in the availability of prior knowledge. During the course of the review, we have discussed a number of studies (Ghatala et al., 1980; Schneider et al., 1989, 1993; Maril et al., 2011) which revealed that a certain amount of performance differences in memory tasks between children and adults can be attributed to adults knowing more about the to-be-remembered information. This was most prominently shown in experiments that compared children and adults, with the children being experts in a domain, whereas the adults are not. In this case, children can outperform adults in a memory task that is closely related to their field of expertise (e.g., Schneider et al., 1993). However, using expert groups of different age to uncover differential memory effects of availability vs. accessibility of prior knowledge comes along with difficulties as well, as there are many uncontrollable sources of differences between the age groups (for example, different histories of gaining expertise; Schneider et al., 1993). An alternative approach for future experiments might be to control the availability of prior knowledge either by carefully assessing the participants’ knowledge of the stimulus material, or, perhaps even better, by experimentally inducing new knowledge structures that are comparable between the different age groups. This experimentally induced knowledge subsequently serves as prior knowledge for the learning of new, related information. An example of this approach was provided in a recent behavioral study (Kumaran, 2013) in which it was shown that prior knowledge about a hierarchy facilitates transitive inference in a new, partly overlapping hierarchy. Participants first acquired a seven-item hierarchy and then performed transitive inference on two new nine-item hierarchies. One of the new nine-item hierarchies contained five items of the old seven-item hierarchy, arranged in their original position in the hierarchy (thus forming a scaffold), the other one was entirely new. Participants performed significantly better in the overlapping hierarchy condition as compared to the entirely new hierarchy. These results suggest that prior knowledge benefits transitive inference performance via a contextual transfer that relates new information to the existing knowledge scaffold.

In sum, studies that use experimentally induced knowledge can greatly benefit our understanding of the effects of prior knowledge on memory, especially for developmental questions They have several advantages: first, they allow careful monitoring of the knowledge available to the participant, thus excluding the possibility that knowledge structures are just not comparable between the two groups. Second, the degree of prior knowledge can be experimentally manipulated, which enables researchers to look at the effects of strength of prior knowledge on memory. Third, the phase during which the participants acquire the knowledge can itself be subject to investigation, which would allow relating learning performance to later memory performance. This would provide a link between work on the emergence of knowledge (e.g., Kumaran et al., 2009, 2012) with work on the effects of prior knowledge on memory (e.g., van Kesteren et al., 2010a,b).

Regarding the ability to access and use one’s prior knowledge for memory, the PFC (both medial and lateral aspects) has been shown to be important. Recent studies on the use of prior knowledge for memory (van Kesteren et al., 2010a,b) point to a key role of the mPFC, which is assumed to act as a resonance detector to determine congruency with prior knowledge. When congruent information is detected, the mPFC might inhibit hippocampal activity and the information is directly integrated into existing knowledge structures (van Kesteren et al., 2012). This reasoning is in line with other claims suggesting that the mPFC provides a “feeling of rightness” during retrieval (Moscovitch and Winocur, 2002) and that it acts as a value integrator during the building up of knowledge (Kumaran et al., 2009). In an attempt to integrate these three conceptualizations, we hypothesize that the mPFC plays a monitoring role for episodic memories by providing an evaluation of fit of both internally and externally generated representations with prior knowledge. In line with a recent review (Nieuwenhuis and Takashima, 2011), we suggest that, based on this evaluation of fit, the mPFC impacts memory processing in the limbic system, especially in the HC.

Besides the mPFC, the lPFC is also important for memory processes related to accessing and using knowledge, including semantic elaboration and strategic encoding and retrieval (e.g., Wagner et al., 1998; Fletcher et al., 2000; Murray and Ranganath, 2007). These functions differ from the ones associated with the mPFC, which might indicate that mPFC and lPFC differ in their contribution to the effects of prior knowledge on memory, depending on the requirements of the memory task at hand. On the one hand, the mPFC might mainly be involved in situations that highlight the congruency of new information with prior knowledge, its task being to evaluate the fit between the target information and expectancies based on prior knowledge. This evaluation is particularly relevant when different behavioral choices are involved in the process of building up the prior knowledge (e.g., choices that entail gains or losses). On the other hand, the lPFC might be involved when there is a strategic/intentional attempt to integrate and relate new knowledge with existing knowledge structures. These conjectures will need to be validated empirically.

Coming back to the development of the use of prior knowledge for memory, the PFC, and in particular its lateral parts, is known to mature late and to reach its full functionality in early adulthood only (Sowell et al., 2003). Therefore, it seems plausible that children do not use their prior knowledge as efficiently as young adults do. This would point to a key role of the PFC in the development of the use of prior knowledge for memory and would also converge with general assumptions about the increasing influence of memory functions that are mediated by the PFC (Shing et al., 2010). Further evidence for this claim has been revealed in behavioral studies that point to an increase in the use of supposedly PFC-driven memory strategies well into adolescence (Schneider et al., 2002; Brehmer et al., 2007; Paz-Alonso et al., 2008; Shing et al., 2008). Accordingly, fMRI and event-related potential (ERP) studies could link the development of memory for context and details, known to particularly require strategic/cognitive control functions, to the maturation of PFC networks (e.g., Cycowicz et al., 2001; Ofen et al., 2007). Taken together, the literature on memory development so far has placed much emphasis on the lateral PFC as a driving force of age-related improvements in memory functioning across childhood.

In addition, we postulate that it is worthwhile taking a closer look on the developmental role that the mPFC may play in supporting knowledge use in memory. It is interesting to note that parts of the mPFC (Shaw et al., 2008) display a protracted structural development. The extent to which this has implications for the functional contributions of the mPFC to the access and use of prior knowledge for memory is yet unknown. If the functional developmental trajectory of parts of the mPFC relevant for knowledge processing resembles the trajectory of the lPFC, an increase in mPFC activation should track the increasing use of knowledge for memory with age. At the same time, due to the fact that children constantly have to build-up new knowledge and update their knowledge structures, the amount of available knowledge increases rapidly during childhood and adolescence. In Piagetian terms, accommodation (changing existing schemas) occurs as long as we continue to learn (Piaget, 1951). Therefore, the involvement of mPFC in building up knowledge may be starting early. Children, although being experts at building up new knowledge, seem to differ from young adults, however, when it comes to accessing and using knowledge strategically. Tackling the factors that limit the strategical use of knowledge by experimentally ensuring the existence of comparable knowledge structures in children and young adults will be highly interesting and relevant to developmental as well as educational scholars. Research into age differences in the use of prior knowledge for memory might prospectively serve to foster learning environments that are better tailored to the way that children acquire knowledge. Given the working hypothesis that children do not use their knowledge as efficiently as young adults, it is tempting to search for ways to foster the use of knowledge in children, for example via training the children on memory strategies (e.g., Brehmer et al., 2007; Shing et al., 2008). An efficient use of knowledge may eliminate age differences in learning and memory performance, as shown in the expertise literature (e.g., Schneider et al., 1993).

In sum, our knowledge of the world is quickly changing and increasing during the first decades of the lifespan. Therefore, a developmental perspective on understanding how the human brain makes use of its accumulated knowledge and how it guides future learning as well as behavior, seems to be highly called for.

## Conflict of Interest Statement

The authors declare that the research was conducted in the absence of any commercial or financial relationships that could be construed as a potential conflict of interest.

## References

[B1] Addis D. R. McAndrews M. P. (2006). Prefrontal and hippocampal contributions to the generation and binding of semantic associations during successful encoding. Neuroimage 33, 1194–1206 10.1016/j.neuroimage.2006.07.039 17023179

[B2] Alba J. W. Hasher L. (1983). Is memory schematic? Psychol. Bull. 93, 203 10.1037/0033-2909.93.2.203

[B3] Andersen P. Morris R. G. M. Amaral D. Bliss T. O’Keefe J. (eds) (2007). The Hippocampus Book. New York: Oxford University Press

[B4] Anderson J. R. (1981). Effects of prior knowledge on memory for new information. Mem. Cognit. 9, 237–246

[B5] Baltes P. B. (1987). Theoretical propositions of life-span developmental psychology: on the dynamics between growth and decline. Dev. Psychol. 23, 611–626 10.1037/0012-1649.23.5.61117158023

[B6] Baltes P. B. Lindenberger U. Staudinger U. M. (2006). “Life-span theory in developmental psychology,” in Handbook of Child Psychology: Vol. 1. Theoretical Models of Human Development, Series edited by Damon W. and Vol. edited by Lerner R. M., 6th ed., (New Jersey: Wiley), 569–664

[B7] Bartlett F. C. (1932). Remembering: A Study in Experimental and Social Psychology. Cambridge: Cambridge University Press

[B8] Behrens T. E. J. Hunt L. T. Woolrich M. W. Rushworth M. F. S. (2008). Associative learning of social value. Nature 456, 245–249 10.1038/nature07538 19005555 PMC2605577

[B9] Binder J. R. Desai R. H. (2011). The neurobiology of semantic memory. Trends Cogn. Sci. 15, 527–536 10.1016/j.tics.2011.10.001 22001867 PMC3350748

[B10] Binder J. R. Desai R. H. Graves W. W. Conant L. L. (2009). Where is the semantic system? A critical review and meta-analysis of 120 functional neuroimaging studies. Cereb. Cortex. 19, 2767–2796 10.1093/cercor/bhp055 19329570 PMC2774390

[B11] Brainerd C. J. Holliday R. E. Reyna V. F. (2004). Behavioral measurement of remembering phenomenologies: so simple a child can do it. Child Dev. 75, 505–522 10.1111/j.1467-8624.2004.00689.x 15056202

[B12] Bransford J. D. Johnson M. K. (1972). Contextual prerequisites for understanding: some investigations of comprehension and recall. J. Verbal Learn. Verbal Behav. 11, 717–726 10.1016/S0022-5371(72)80006-9

[B13] Brehmer Y. Li S.-C. Müller V. von Oertzen T. Lindenberger U. (2007). Memory plasticity across the life span: uncovering children’s latent potential. Dev. Psychol. 43, 465–478 10.1037/0012-1649.43.2.465 17352553

[B14] Bunge S. A. Dudukovic N. M. Thomason M. E. Vaidya C. J. Gabrieli J. D. E. (2002). Immature frontal lobe contributions to cognitive control in children. Neuron 33, 301–311 10.1016/S0896-6273(01)00583-9 11804576 PMC4535916

[B15] Chai X. J. Ofen N. Jacobs L. F. Gabrieli J. D. E. (2010). Scene complexity: influence on perception, memory, and development in the medial temporal lobe. Front. Hum. Neurosci. 4, 10.3389/fnhum.2010.00021 20224820 PMC2835514

[B16] Cohen G. Burke D. M. (1993). Memory for proper names: a review. Memory 1, 249–263 10.1080/09658219308258237 7584271

[B17] Craik F. I. M. Bialystok E. (2006). Cognition through the lifespan: mechanisms of change. Trends Cogn. Sci. 10, 131–138 10.1016/j.tics.2006.01.007 16460992

[B18] Craik F. I. M. Lockhart R. S. (1972). Levels of processing: a framework for memory research. J. Verbal Learn. Verbal Behav. 11, 671–684 10.1016/S0022-5371(72)80001-X

[B19] Craik F. I. M. Tulving E. (1975). Depth of processing and the retention of words in episodic memory. J. Exp. Psychol. Gen. 104, 268–294 10.1037/0096-3445.104.3.268

[B20] Cycowicz Y. M. Friedman D. Snodgrass J. G. Duff M. (2001). Recognition and source memory for pictures in children and adults. Neuropsychologia 39, 255–267 10.1016/S0028-3932(00)00108-1 11163604

[B21] Deese J. (1959). On the prediction of occurrence of particular verbal intrusions in immediate recall. J. Exp. Psychol. 58, 17–22 10.1037/h0046671 13664879

[B22] Diamond A. (2002). “Normal development of prefrontal cortex from birth to young adulthood: cognitive functions, anatomy, and biochemistry,” in Principles of Frontal Lobe Function, eds Stuss D. T. Knight R. T. (New York: Oxford University Press), 466–503

[B23] Duncan K. Curtis C. Davachi L. (2009). Distinct memory signatures in the hippocampus: intentional states distinguish match and mismatch enhancement signals. J. Neurosci. 29, 131–139 10.1523/JNEUROSCI.2998-08.2009 19129391 PMC2789241

[B24] Dunlosky J. Hunt R. R. Clark E. (2000). Is perceptual salience needed in explanations of the isolation effect? J. Exp. Psychol. Learn. Mem. Cogn. 26, 649–657 10.1037/0278-7393.26.3.649 10855423

[B25] Ellenbogen J. M. Hu P. T. Payne J. D. Titone D. Walker M. P. (2007). Human relational memory requires time and sleep. Proc. Natl. Acad. Sci. U.S.A. 104, 7723–7728 10.1073/pnas.0700094104 17449637 PMC1863467

[B26] Fletcher P. C. Shallice T. Dolan R. (2000). Sculpting the response space – an account of left prefrontal activation at encoding. Neuroimage 12, 404–417 10.1006/nimg.2000.0633 10988034

[B27] Frankland P. W. Bontempi B. (2005). The organization of recent and remote memories. Nat. Rev. Neurosci. 6, 119–130 10.1038/nrn1607 15685217

[B28] Gais S. Albouy G. Boly M. Dang-Vu T. T. Darsaud A. Desseilles M. (2007). Sleep transforms the cerebral trace of declarative memories. Proc. Natl. Acad. Sci. U.S.A. 104, 18778–18783 10.1073/pnas.0705454104 18000060 PMC2141853

[B29] Geis M. F. Hall D. M. (1978). Encoding and congruity in children’s incidental memory. Child Dev. 49, 857–861 10.2307/1128256

[B30] Ghatala E. S. Carbonari J. P. Bobele L. Z. (1980). Developmental changes in incidental memory as a function of processing level, congruity, and repetition. J. Exp. Child Psychol. 29, 74–87 10.1016/0022-0965(80)90092-2 7354268

[B31] Ghetti S. DeMaster D. M. Yonelinas A. P. Bunge S. A. (2010). Developmental differences in medial temporal lobe function during memory encoding. J. Neurosci. 30, 9548–9556 10.1523/JNEUROSCI.3500-09.2010 20631183 PMC2937832

[B32] Gilboa A. Alain C. Stuss D. T. Melo B. Miller S. Moscovitch M. (2006). Mechanisms of spontaneous confabulations: a strategic retrieval account. Brain 129, 1399–1414 10.1093/brain/awl093 16638795

[B33] Gogtay N. Nugent T. F. Herman D. H. Ordonez A. Greenstein D. Hayashi K. M. (2006). Dynamic mapping of normal human hippocampal development. Hippocampus 16, 664–672 10.1002/hipo.20193 16826559

[B34] Golarai G. Ghahremani D. G. Whitfield-Gabrieli S. Reiss A. Eberhardt J. L. Gabrieli J. D. E. (2007). Differential development of high-level visual cortex correlates with category-specific recognition memory. Nat. Neurosci. 10, 512–522 17351637 10.1038/nn1865 PMC3660101

[B35] Hasher L. Tonev S. T. Lustig C. Zacks R. T. (2001). “Inhibitory control, environmental support, and self-initiated processing in aging,” in Perspectives on Human Memory and Cognitive Aging: Essays in Honour of Fergus Craik, eds Naveh-Benjamin M. Moscovitch M. Roediger H. L. (New York: Psychology Press), 286–297

[B36] Hunt R. R. Lamb C. A. (2001). What causes the isolation effect? J. Exp. Psychol. Learn. Mem. Cogn. 27, 1359–1366 10.1037/0278-7393.27.6.1359 11713872

[B37] Kapur S. Craik F. I. Tulving E. Wilson A. A. Houle S. Brown G. M. (1994). Neuroanatomical correlates of encoding in episodic memory: levels of processing effect. Proc. Natl. Acad. Sci. U.S.A. 91, 2008–2011 10.1073/pnas.91.6.2008 8134340 PMC43298

[B38] Köhler W. von Restorff H. (1937). Analyse von Vorgängen im Spurenfeld. Psychol. Forsch. 21, 56–112 10.1007/BF02441202

[B39] Kroes M. C. W. Fernández G. (2012). Dynamic neural systems enable adaptive, flexible memories. Neurosci. Biobehav. Rev. 36, 1646–1666 10.1016/j.neubiorev.2012.02.014 22874579

[B40] Kumaran D. (2013). Schema-driven facilitation of new hierarchy learning in the transitive inference paradigm. Learn. Mem. 20, 388–394 10.1101/lm.030296.113 23782509 PMC3687256

[B41] Kumaran D. McClelland J. L. (2012). Generalization through the recurrent interaction of episodic memories: a model of the hippocampal system. Psychol. Rev. 119, 573–616 10.1037/a0028681 22775499 PMC3444305

[B42] Kumaran D. Melo H. L. Duzel E. (2012). The emergence and representation of knowledge about social and nonsocial hierarchies. Neuron 76, 653–666 10.1016/j.neuron.2012.09.035 23141075 PMC3580285

[B43] Kumaran D. Summerfield J. J. Hassabis D. Maguire E. A. (2009). Tracking the emergence of conceptual knowledge during human decision making. Neuron 63, 889–901 10.1016/j.neuron.2009.07.030 19778516 PMC2791172

[B44] Lavenex P. Lavenex P. B. (2013). Building hippocampal circuits to learn and remember: insights into the development of human memory. Behav. Brain Res. 254, 8–21 10.1016/j.bbr.2013.02.007 23428745

[B45] Li S.-C. Lindenberger U. Hommel B. Aschersleben G. Prinz W. Baltes P. B. (2004). Transformations in the couplings among intellectual abilities and constituent cognitive processes across the life span. Psychol. Sci. 15, 155–163 10.1111/j.0956-7976.2004.01503003.x 15016286

[B46] Maguire E. A. Frith C. D. Morris R. G. M. (1999). The functional neuroanatomy of comprehension and memory: the importance of prior knowledge. Brain 122, 1839–1850 10.1093/brain/122.10.1839 10506087

[B47] Maril A. Avital R. Reggev N. Zuckerman M. Sadeh T. Ben Sira L. (2011). Event congruency and episodic encoding: a developmental fMRI study. Neuropsychologia 49, 3036–3045 10.1016/j.neuropsychologia.2011.07.004 21777596

[B48] Marr D. (1971). Simple memory: a theory for archicortex. Philos. Trans. R. Soc. Lond. B Biol. Sci. 262, 23–81 10.1098/rstb.1971.0078 4399412

[B49] McClelland J. L. McNaughton B. L. O’Reilly R. C. (1995). Why there are complementary learning systems in the hippocampus and neocortex: insights from the successes and failures of connectionist models of learning and memory. Psychol. Rev. 102, 419–457 10.1037/0033-295X.102.3.419 7624455

[B50] McKenzie S. Robinson N. T. M. Herrera L. Churchill J. C. Eichenbaum H. (2013). Learning causes reorganization of neuronal firing patterns to represent related experiences within a hippocampal schema. J. Neurosci. 33, 10243–10256 10.1523/JNEUROSCI.0879-13.2013 23785140 PMC3685831

[B51] Metzger R. L. Warren A. R. Shelton J. T. Price J. Reed A. W. Williams D. (2008). Do children “DRM” like adults? False memory production in children. Dev. Psychol. 44, 169–181 10.1037/0012-1649.44.1.169 18194015

[B52] Mitchell K. J. Johnson M. K. (2009). Source monitoring 15 years later: what have we learned from fMRI about the neural mechanisms of source memory? Psychol. Bull. 135, 638–677 10.1037/a0015849 19586165 PMC2859897

[B53] Moscovitch M. Craik F. I. M. (1976). Depth of processing, retrieval cues, and uniqueness of encoding as factors in recall. J. Verbal Learn. Verbal Behav. 15, 447–458

[B54] Moscovitch M. Winocur G. (2002). “The frontal cortex and working with memory,” in Principles of Frontal Lobe Function, eds Stuss D. T. Knight R. T. (New York: Oxford University Press), 188–209

[B55] Murray L. J. Ranganath C. (2007). The dorsolateral prefrontal cortex contributes to successful relational memory encoding. J. Neurosci. 27, 5515–5522 10.1523/JNEUROSCI.0406-07.2007 17507573 PMC6672342

[B56] Nadel L. Moscovitch M. (1997). Memory consolidation, retrograde amnesia and the hippocampal complex. Curr. Opin. Neurobiol. 7, 217–227 10.1016/S0959-4388(97)80010-4 9142752

[B57] Nieuwenhuis I. L. C. Takashima A. (2011). The role of the ventromedial prefrontal cortex in memory consolidation. Behav. Brain Res. 218, 325–334 10.1016/j.bbr.2010.12.009 21147169

[B58] Northoff G. Bermpohl F. (2004). Cortical midline structures and the self. Trends Cogn. Sci. 8, 102–107 10.1016/j.tics.2004.01.004 15301749

[B59] Ofen N. (2012). The development of neural correlates for memory formation. Neurosci. Biobehav. Rev. 36, 1708–1717 22414608 10.1016/j.neubiorev.2012.02.016 PMC3437984

[B60] Ofen N. Kao Y.-C. Sokol-Hessner P. Kim H. Whitfield-Gabrieli S. Gabrieli J. D. E. (2007). Development of the declarative memory system in the human brain. Nat. Neurosci. 10, 1198–1205 10.1038/nn1950 17676059

[B61] Paz-Alonso P. M. Ghetti S. Donohue S. E. Goodman G. S. Bunge S. A. (2008). Neurodevelopmental correlates of true and false recognition. Cereb. Cortex 18, 2208–2216 10.1093/cercor/bhm246 18203693 PMC2517100

[B62] Piaget J. (1929). The Child’s Conception of the World, trans. Tomlinson J. Thomlinson A. (New York: Harcourt Brace).

[B63] Piaget J. (1951). Play, Dreams and Imitation in Childhood. London: Routledge

[B64] Ranganath C. Johnson M. K. D’Esposito M. (2000). Left anterior prefrontal activation increases with demands to recall specific perceptual information. J. Neurosci. 20, RC108 11069977 10.1523/JNEUROSCI.20-22-j0005.2000 PMC6773176

[B65] Reber P. J. Siwiec R. M. Gitelman D. R. Parrish T. B. Mesulam M. M. Paller K. A. (2002). Neural correlates of successful encoding identified using functional magnetic resonance imaging. J. Neurosci. 22, 9541–9548 12417678 10.1523/JNEUROSCI.22-21-09541.2002 PMC6758020

[B66] Roediger H. L. McDermott K. B. (1995). Creating false memories: remembering words not presented in lists. J. Exp. Psychol. Learn. Mem. Cogn. 21, 803–814 10.1037/0278-7393.21.4.803

[B67] Rojahn K. Pettigrew T. F. (1992). Memory for schema-relevant information: a meta-analytic resolution. Br. J. Soc. Psychol. 31, 81–109 10.1111/j.2044-8309.1992.tb00958.x 1535823

[B68] Schneider W. Gruber H. Gold A. Opwis K. (1993). Chess expertise and memory for chess positions in children and adults. J. Exp. Child Psychol. 56, 328–349 10.1006/jecp.1993.1038 8301242

[B69] Schneider W. Knopf M. Stefanek J. (2002). The development of verbal memory in childhood and adolescence: findings from the Munich longitudinal study. J. Educ. Psychol. 94, 751–761 10.1037/0022-0663.94.4.751

[B70] Schneider W. Körkel J. Weinert F. E. (1989). Domain-specific knowledge and memory performance: a comparison of high- and low-aptitude children. J. Educ. Psychol. 81, 306–312 10.1037/0022-0663.81.3.306

[B71] Schulman A. I. (1974). Memory for words recently classified. Mem. Cognit. 2, 47–52 10.3758/BF03197491 24214698

[B72] Shaw P. Kabani N. J. Lerch J. P. Eckstrand K. Lenroot R. Gogtay N. (2008). Neurodevelopmental trajectories of the human cerebral cortex. J. Neurosci. 28, 3586–3594 10.1523/JNEUROSCI.5309-07.2008 18385317 PMC6671079

[B73] Shing Y. L. Werkle-Bergner M. Brehmer Y. Müller V. Li S.-C. Lindenberger U. (2010). Episodic memory across the lifespan: the contributions of associative and strategic components. Neurosci. Biobehav. Rev. 34, 1080–1091 19896974 10.1016/j.neubiorev.2009.11.002

[B74] Shing Y. L. Werkle-Bergner M. Li S.-C. Lindenberger U. (2008). Associative and strategic components of episodic memory: a life-span dissociation. J. Exp. Psychol. Gen. 137, 495–513 10.1037/0096-3445.137.3.495 18729712

[B75] Simons J. S. Spiers H. J. (2003). Prefrontal and medial temporal lobe interactions in long-term memory. Nat. Rev. Neurosci. 4, 637–648 10.1038/nrn1178 12894239

[B76] Sloutsky V. M. Fisher A. V. (2004). When development and learning decrease memory: evidence against category-based induction in children. Psychol. Sci. 15, 553–558 10.1111/j.0956-7976.2004.00718.x 15271001

[B77] Smith R. E. Hunt R. R. (1998). Presentation modality affects false memory. Psychon. Bull. Rev. 5, 710–715 10.1037/a0022217 21261425

[B78] Sowell E. R. Peterson B. S. Thompson P. M. Welcome S. E. Henkenius A. L. Toga A. W. (2003). Mapping cortical change across the human life span. Nat. Neurosci. 6, 309–315 10.1038/nn1008 12548289

[B79] Squire L. R. (1992). Memory and the hippocampus: a synthesis from findings with rats, monkeys, and humans. Psychol. Rev. 99, 195–231 10.1037/0033-295X.99.3.582 1594723

[B80] Stangor C. McMillan D. (1992). Memory for expectancy-congruent and expectancy-incongruent information: a review of the social and social developmental literatures. Psychol. Bull. 111, 42–61 10.1037/0033-2909.111.1.42

[B81] Staresina B. P. Gray J. C. Davachi L. (2009). Event congruency enhances episodic memory encoding through semantic elaboration and relational binding. Cereb. Cortex 19, 1198–1207 10.1093/cercor/bhn165 18820289 PMC2665161

[B82] Takashima A. Nieuwenhuis I. L. C. Jensen O. Talamini L. M. Rijpkema M. Fernandez G. (2009). Shift from hippocampal to neocortical centered retrieval network with consolidation. J. Neurosci. 29, 10087–10093 10.1523/JNEUROSCI.0799-09.2009 19675242 PMC6664975

[B83] Takashima A. Petersson K. M. Rutters F. Tendolkar I. Jensen O. Zwarts M. J. (2006). Declarative memory consolidation in humans: a prospective functional magnetic resonance imaging study. Proc. Natl. Acad. Sci. U.S.A. 103, 756–761 10.1073/pnas.0507774103 16407110 PMC1334654

[B84] Treves A. Rolls E. T. (1992). Computational constraints suggest the need for two distinct input systems to the hippocampal CA3 network. Hippocampus 2, 189–199 10.1002/hipo.450020209 1308182

[B85] Tse D. Langston R. F. Kakeyama M. Bethus I. Spooner P. A. Wood E. R. (2007). Schemas and memory consolidation. Science 316, 76–82 10.1126/science.1135935 17412951

[B86] Tse D. Takeuchi T. Kakeyama M. Kajii Y. Okuno H. Tohyama C. (2011). Schema-dependent gene activation and memory encoding in neocortex. Science 333, 891–895 10.1126/science.1205274 21737703

[B87] Tulving E. (1972). “Episodic and semantic memory,” in Organization of Memory, eds Tulving E. Donaldson W. (New York: Academic Press), 381–402

[B88] van Kesteren M. T. R. Beul S. F. Takashima A. Henson R. N. Ruiter D. J. Fernández G. (2013). Differential roles for medial prefrontal cortex and medial temporal cortices in schema-dependent encoding: from congruent to incongruent. Neuropsychologia. 10.1016/j.neuropsychologia.2013.05.027 23770537

[B89] van Kesteren M. T. R. Fernández G. Norris D. G. Hermans E. J. (2010a). Persistent schema-dependent hippocampal-neocortical connectivity during memory encoding and postencoding rest in humans. Proc. Natl. Acad. Sci. U.S.A. 107, 7550–7555 10.1073/pnas.0914892107 20363957 PMC2867741

[B90] van Kesteren M. T. R. Rijpkema M. Ruiter D. J. Fernández G. (2010b). Retrieval of associative information congruent with prior knowledge is related to increased medial prefrontal activity and connectivity. J. Neurosci. 30, 15888–15894 10.1523/JNEUROSCI.2674-10.2010 21106827 PMC6633736

[B91] van Kesteren M. T. R. Ruiter D. J. Fernández G. Henson R. N. (2012). How schema and novelty augment memory formation. Trends Neurosci. 35, 211–219 10.1016/j.tins.2012.02.001 22398180

[B92] Vargha-Khadem F. Gadian D. G. Watkins K. E. Connelly A. Van Paesschen W. Mishkin M. (1997). Differential effects of early hippocampal pathology on episodic and semantic memory. Science 277, 376–380 10.1126/science.277.5324.376 9219696

[B93] Wagner A. D. Schacter D. L. Rotte M. Koutstaal W. Maril A. Dale A. M. (1998). Building memories: remembering and forgetting of verbal experiences as predicted by brain activity. Science 281, 1188–1191 10.1126/science.281.5380.1188 9712582

[B94] Wang S.-H. Morris R. G. M. (2010). Hippocampal-neocortical interactions in memory formation, consolidation, and reconsolidation. Annu. Rev. Psychol. 61, 49–79 10.1146/annurev.psych.093008.100523 19575620

[B95] Werkle-Bergner M. Müller V. Li S.-C. Lindenberger U. (2006). Cortical EEG correlates of successful memory encoding: implications for lifespan comparisons. Neurosci. Behav. Rev. 30, 839–854 16904180 10.1016/j.neubiorev.2006.06.009

[B96] Yamashita K. I. Hirose S. Kunimatsu A. Aoki S. Chikazoe J. Jimura K. (2009). Formation of long-term memory representation in human temporal cortex related to pictorial paired associates. J. Neurosci. 29, 10335–10340 10.1523/JNEUROSCI.1328-09.2009 19692607 PMC6665779

[B97] Yassa M. A. Stark C. (2011). Pattern separation in the hippocampus. Trends Neurosci. 34, 515–525 10.1016/j.tins.2011.06.006 21788086 PMC3183227

[B98] Zeithamova D. Preston A. R. (2010). Flexible memories: differential roles for medial temporal lobe and prefrontal cortex in cross-episode binding. J. Neurosci. 30, 14676–14684 10.1523/JNEUROSCI.3250-10.2010 21048124 PMC6633616

[B99] Zelazo P. D. Craik F. I. M. Booth L. (2004). Executive function across the life span. Acta Psychol. 115, 167–183 10.1016/j.actpsy.2003.12.005 14962399

